# Cytomegalovirus viral and antibody correlates in young children

**DOI:** 10.1186/1756-0500-7-776

**Published:** 2014-11-03

**Authors:** Sheila C Dollard, Harry Keyserling, Kay Radford, Minal M Amin, Jennifer Stowell, Jörn Winter, D Scott Schmid, Michael J Cannon, Terri B Hyde

**Affiliations:** Centers for Disease Control and Prevention, 1600 Clifton Rd NE, Atlanta, GA 30333 USA; Emory University Department of Pediatric Infectious Diseases, Atlanta, GA USA

**Keywords:** Cytomegalovirus, Viral load, Serology, Avidity

## Abstract

**Background:**

Young, healthy children shedding cytomegalovirus (CMV) in urine and saliva appear to be the leading source of CMV in primary infection of pregnant women.

**Findings:**

We screened 48 children 6 months – 5 years old for CMV IgG and measured levels of CMV IgG, IgM and IgG avidity antibodies, frequency of CMV shedding, and viral loads in blood, urine, and saliva. Thirteen of the 48 children (27%) were CMV IgG positive, among whom 3 were also CMV IgM positive with evidence of recent primary infection. Nine of the 13 seropositive children (69%) were shedding 10^2^-10^5^ copies/ml of CMV DNA in one or more bodily fluid. Among seropositive children, low IgG antibody titer (1:20–1:80) was associated with the absence of shedding (p = 0.014), and enrollment in daycare was associated with the presence of CMV shedding (p = 0.037).

**Conclusions:**

CMV antibody profiles correlated with CMV shedding. The presence of CMV IgM more often represents primary infection in children than in adults. Correlating antibodies with primary infection and viral shedding in healthy children adds to the understanding of CMV infection in children that can inform the prevention of CMV transmission to pregnant women.

## Background

Infection with human cytomegalovirus (CMV) is typically harmless for children and adults but can have serious consequences for immunocompromised persons and developing fetuses. In the United States, 0.5 - 1% (20,000 - 40,000) of infants are born with congenital CMV infection each year and approximately 20% of them (4,000 - 8,000) develop permanent disabilities such as hearing loss, visual impairment, and mental retardation [1, 2]. CMV infection occurs through close interpersonal contact with infected bodily fluids and usually confers no symptoms. Young children who become infected often shed CMV in urine and saliva for a year or more and are thought to be the leading source of CMV for primary infection in women of reproductive age [3–7]. Several studies have shown shedding of CMV virus in young children mainly by performing viral culture on urine or saliva [7–9]. However most studies tested a single specimen type and very few studies measured virus in blood or included serology to identify the proportion of children with CMV infection that were shedding.

The main objective of this study was to examine CMV antibody profiles in young children as they relate to virus levels across urine, saliva, and blood. This report provides new information on the frequency of shedding among CMV seropositive children, including identification of primary infection, and compares CMV viral loads across fluids collected at the same time. Knowledge of associations between immune-response parameters and CMV shedding and viral loads in bodily fluids may enhance understanding of natural CMV infection in toddlers and inform prevention measures for women of reproductive age.

## Findings

### Methods

This study was approved by the Institutional Review Boards (IRB) of the CDC and Emory University (CDC IRB study protocol #4630). Written informed consent for participation in the study was obtained from parents or guardians for the children in this study. We enrolled children between the ages of 6 and 60 months who were having blood drawn for other purposes at four Emory University outpatient pediatric clinics. The enrollment questionnaire collected information on age, sex, attendance in daycare (institutional or home-based), race/ethnicity, and type of health insurance as a marker for socioeconomic status (SES). We did not have information on the timing of CMV infection for any children since CMV is typically an asymptomatic infection. We did not have information on whether any of the 13 seropositive children may have been born with CMV, but this would have low probability since the congenital CMV infection rate is approximately 0.65% (1 per 154 infants) [2].

There was a one-time collection of 3 bodily fluids: whole blood, urine, and saliva. Whole blood was collected through venipuncture into EDTA Vacuatiners (Becton-Dickinson, New Jersey, USA) with a portion processed for antibody testing. CMV IgG and IgM status were determined by VIDAS. End-point titer was performed by immunofluorescense assay (IFA) (MBL-Bion, Des Plaines, IL) because the IFA requires far less specimen volume than the VIDAS. IgG avidity was measured with 3 tests (Euroimmun, Vidia, and VIDAS) because of reports of low concordance among commercial CMV avidity tests [10]. Interpretation of the Euroimmun and Vidia tests followed manufacturer’s instructions. Interpretation of the VIDAS was adjusted to provide improved sensitivity [11–13]. Final avidity determination was based on agreement of two or more tests results. Saliva was collected with the Oracol foam swab (Malvern Medical Developments Ltd, Worcester, England). Urine was collected by having children urinate into a specimen cup with their mothers helping as needed. Total DNA was extracted from whole blood, urine, and saliva using the MagNAPure with DNA extraction kits (Roche Diagnostics) followed by Taqman-based PCR [14, 15]. Viral copy numbers were normalized to the input volume for each specimen so that viral loads were comparable across the 3 bodily fluids. Statistical significance for various associations was calculated using Fisher’s exact test (two-tailed, 95% confidence interval). Differences in CMV viral load by specimen type were compared using a two-sided t-test with unequal variances.

### Results

Total enrollment was 48 children; 54% were male and 46% were female; 18% were 6–11 months old, 74% were 1–2 years old, and 8% were 3–4 years old. Blood was collected from all 48 enrolled children, saliva was collected from 46/48 (96%) children, and urine was collected from only 26/48 (54%) children because many of them were unable to produce urine during the 30 minute clinic visit. VIDAS determinations for IgG and IgM were the same as those by IFA. CMV IgG was detected in 13/48 (27%) of the children (Table 1) all of whom were over 12 months old, eliminating the possible interference of maternal antibody. Their racial/ethnic makeup and SES are shown on Table 1. Three of the IgG-positive children were CMV IgM-positive. Among IgM positive sera, two were low IgG avidity and one was low-intermediate IgG avidity (Table 1), indicating that all three represented primary infection. Two out of 3 IgM-positive sera had virus in bodily fluids that might suggest a correlation between the presence of IgM and the presence of shedding. Avidity results for the 3 commercial tests were in complete agreement for 10/13 sera and had imperfect agreement for the following 3 borderline sera: H1 had two intermediate values and one high value; D6 had two low values and one intermediate value; D8 had two intermediate values and one low value. Results of the Euroimmun test were in the majority result for all sera and are shown in Table 1. Ten of the 13 antibody-positive children (69%) had CMV DNA in one or more bodily fluid. Shedding in urine is not shown on Table 1 because of the low number of specimens collected. The prevalence of CMV DNA was 62% (8/13) for saliva and 46% (6/13) for blood (Table 1). Geometric mean viral loads were 3727 copies/ml for urine, 4449 copies/ml for saliva, and 172 copies/ml for blood. The difference in mean viral loads was statistically significant for saliva and blood (p = 0.01). There were too few urine samples to include them in analysis. The 3 positive urine samples had the following viral loads: (Table 1 child numbers) D4: 27,756 copies/ml, D6: 667 copies/ml, and D7: 2,798 copies/ml. None of the 35 CMV IgG-negative children had CMV DNA in any bodily fluid. Enrollment in daycare showed a significant association with CMV viral shedding (p = 0.037). Low IgG antibody titer, defined as endpoint titer 1:20–1:80 (range 1:20 – 1:1280), was associated with the absence of shedding (p = 0.014). Age, race/ethnicity, and socioeconomic status showed no association with CMV antibody positivity or shedding; however, the small sample size had limited ability to show associations.

**Table 1:** **CMV antibody titers, IgG avidity status, and viral loads for the CMV seropositive children according to daycare attendance**

Child home (H) daycare (D)	Race/ ethnicity*	Socio-economic status	IgG titer	IgG VIDAS UA/ml >5 pos	IgM titer	IgG avidity index**	IgG avidity status	Saliva cop/ml	Blood cop/ml
H1	Black	Low	1:20	13	-	48.32	Interm	-	-
H2	Black	Low	1:20	7	-	61.89	High	-	-
H3	Black	Med/High	1:20	11	-	65.36	High	718	133
H4	White-NH	Med/High	1:1280	154	-	79.00	High	994	84
H5	White-His	Low	1:40	12	-	78.72	High	-	-
D1	White-His	Low	1:320	91	1:160	25.92	Low	7,212	550
D2	Black	Low	1:320	64	-	63.69	High	3,130	287
D3	White-NH	Med/High	1:320	74	-	72.87	High	92,056	-
D4	White-NH	Low	1:160	48	-	73.82	High	24,722	207
D5	Black	Low	1:160	60	-	76.77	High	17,181	-
D6	Black	Med/High	1:1280	110	1:20	35.21	Low	-	92
D7	Black	Med/High	1:320	41	-	72.33	High	244	-
D8	White-His	Low	1:80	37	1:20	42.24	Interm	-	-

*NH = non-Hispanic; His = Hispanic. **Cutoff values for Euroimmun CMV avidity test: <40 = low avidity, 40–60 = intermediate avidity, >60 = high avidity.

## Discussion

The main objective of this study was to measure CMV viral loads in urine, saliva, and blood compared to antibody profiles to identify possible correlates between serology and shedding. Many studies have reported CMV shedding in urine and saliva of young children. The unique aspect of our study was the inclusion of CMV serology. We observed a strong correlation between CMV shedding (the presence of CMV DNA in any bodily fluid) and enrollment in daycare previously reported that indicates that spread of CMV occurs in daycare centers [16, 17]. New and suggestive findings include: (1) A significant correlation between low IgG titers and the absence of CMV shedding; (2) High association between IgM-positive status and recent primary infection, in contrast to CMV IgM in adults for which less than 20% is estimated to represent recent primary infection [11, 18]. (3) Prolonged CMV viremia; among nine children with evidence of infection more than 4 months prior, four (44%) were still viremic. This is consistent with long periods of CMV shedding in children and contrasts with adults who show much shorter periods of CMV viremia following primary infection [19].

Our study had the main limitation of small numbers due to lower enrollment than expected and can only provide a basis for future studies. One potential study could establish whether CMV IgM reactivity in young children usually indicates primary CMV infection. This would be expected since younger people experience more primary than non-primary infection, and is supported by a US population based study showing that the presence of CMV IgM in adolescents is far more associated with low IgG avidity than CMV IgM in adults [11]. Among the 13 seropositive children, 9 (69%) had CMV in urine, saliva, or blood. This is likely an underestimate for CMV shedding for two reasons, because we tested a single time point for a virus that can shed intermittently [7], and because urine is a common vehicle for CMV shedding [7] and was not available for half the children. Larger studies may also explore possible correlations between CMV antibody titer and CMV shedding.

## Conclusions

Our study found that 9 of 13 CMV seropositive children (69%) were shedding virus in one or more bodily fluid. Extended periods of CMV shedding in young children have been reported [7] and are in contrast to shorter duration of CMV shedding in adults [20] which may explain the major role of young children in CMV transmission. Few studies on CMV in children have included serology and our study suggests serology may provide useful information beyond infection status regarding the timing of infection and shedding correlates: CMV IgM was more associated with primary infection compared to adults, and children with low IgG titers were less likely to be IgM-positive or to be shedding, possibly reflecting more elapsed time since primary infection. Noninvasive determination CMV serostatus of young children, for example from a finger prick, may contribute useful information to infection prevention measures. Larger studies would be necessary to verify relationships between serology and viral shedding suggested by the present study that may aid the understanding and prevention of CMV transmission to pregnant women.
